# Study of Drilling Process by Cooling Compressed Air in Reinforced Polyether-Ether-Ketone

**DOI:** 10.3390/ma13081965

**Published:** 2020-04-22

**Authors:** Rosario Domingo, Beatriz de Agustina, Marta María Marín

**Affiliations:** Department of Construction and Manufacturing Engineering, Universidad Nacional de Educación a Distancia (UNED), C/Juan del Rosal 12, E–28040 Madrid, Spain; bdeagustina@ind.uned.es (B.d.A.); mmarin@ind.uned.es (M.M.M.)

**Keywords:** drilling, cooling compressed air, thrust force, energy, material removed rate, PEEK-GF30, multi-response optimization, sustainable manufacturing

## Abstract

This study is focused on the application of a cooling compressed air system in drilling processes; this environmentally friendly technique allows removing material at very low temperatures, approximately up to −22 °C in the cutting area. The main goals are to find the most improve cutting conditions with less energy consumption, for the drilling of reinforced polyether-ether-ketone with glass fiber at 30% (PEEK-GF30) with cooling compressed air by a Ranque-Hilsch vortex tube, and to find a balance between environmental conditions and adequate process performance. Drilling tests were carried out on plates of PEEK-GF30 to analyze the influence of cutting parameters and environmental temperature (–22, 0 and 22 °C) on variables such as thrust forces, energy and material removed rate by the use of statistical methods; analysis of variance, analysis of means, response surface, and desirability function were employed to identify the optimum region that provides the most improved values of the aforementioned variables. Drill bit diameter was also analyzed to determine the quality of drilled holes. During the drilling processes, force signals were detected by a piezoelectric dynamometer connected to multichannel amplifier and a pyrometer was used to control the temperature. The diameters of the drilled holes were measured by a coordinate measuring machine. Cooling compressed air can be considered an adequate technique to improve the results from an environmental and efficient perspective; in particular, the maximum desirability function was found at a spindle speed of 7000 rpm, a feedrate of 1 mm/rev and a temperature close to −22 °C.

## 1. Introduction

Over the past decade, within manufacturing industries there has been an increasing interest in researching techniques that allow the performance of processes with high efficiency under sustainable environments. In fact, different systems have been applied in manufacturing facilities to identify the most improved parameters to combine both objectives; in some cases, the effects on the organization of production system by the implementation of lean techniques [1] and in others, the effects on the manufacturing process where the improved selection of parameters can reduce CO_2_ emissions [2]. The machining is the most used manufacturing process in the industry due to its versatility [3], so studies in this field can be relevant. In this context, several methods have been applied to achieve both objectives, efficiency and sustainability, to the most possible extent. One of them is dry machining or machining without the use of any cutting fluid. Krolczyk et al. [4] have carried out an extensive literature review on four ecological methods in machining of difficult to-cut metals, in particular dry machining, minimum quantity lubrication (MQL)/minimum quantity cooling lubrication (MQCL), cryogenic cooling, high-pressure cooling and biodegradable oils; they found that dry machining is the most sustainable procedure with respect to others, despite the high temperature generated during the machining operation, in particular in operations such as drilling; besides, these authors pointed out the non-use of cryogenic cooling in industry despite the benefits of this procedure; this is the combination of high productivity as well as low cost and energy. Sen et al. [5], in their literature review focussed on metal cutting, collected results from life cycle assessment (LCA) models for different cooling techniques, showing that MQL and dry machining have the minimum negative effects respect to techniques such as flood lubrication, MQL, cryogenic cooling with CO_2_, cryogenic cooling with LN_2_ and cryogenic cooling with MQL. However, machining under such dry conditions causes excessive temperature rise at the interface between workpiece and tool and, in general, an increase in strains. These undesirable effects have been determined by the analysis of forces and strains [6] or considering the influence of tribology [7]; in both cases finite element models (FEMs) were used in the orthogonal cut of titanium alloys, so the application of new cooling techniques can be explored. Cooling technologies [8], such as cryogenic machining with liquid nitrogen and the application of cooling compressed air to the cutting zone during the machining process by means of a Ranque-Hilsch vortex tube have been identified as possible environmentally friendly procedures. These cooling systems are particularly interesting under dry cutting conditions; in this way, cutting fluids are avoided. Goindi and Sarkar [9] identified cooling compressed air obtained by vortex tube separation as a method to analyze into sustainable machining, as a variant of dry machining and also as an element to consider within the MQL. Moreover, the use of cooling compressed air systems in machining processes requires less investment than cryogenic machining and they are easier to employ on an industrial scale. In addition, although the MQL procedure is convenient from the point of view of sustainability, the use of lubricants could not be suitable in polymeric materials due to its possible absorption by the composite.

In fact, the application of cooling compressed air during the machining process is being studied and some researches can show its suitability as environmentally friendly procedure. Jozić et al. [10] developed an experimental study, in which several machining aspects were analyzed in the milling process of a certain steel with this procedure at −34 °C under dry cutting conditions and also with the use of cutting fluid; it was found the most improved solution (less surface roughness, cutting forces, flank wear and more volume of removed material) in case of the application of cooling air during the process. In addition, regarding the milling processes, Perri et al. [11] analyzed the effect of cooling air on the milling tool, using simulation models by FEM and experimental contrast and verifying important difference of temperatures reached respect to the procedure carried out without cooling air. On the other hand, Nor Khairusshima et al. [12] studied the quality and the tool wear in the milling of the carbon fiber reinforced plastic with cooling air at −10 °C; they pointed out that the tool wear and delamination factor were improved at high cutting speeds. Domingo et al. [13] analyzed the effects of cooling air on reinforced and unreinforced polyamides during the tapping, finding that an adequate procedure at −18 °C provided improved results with respect to the values of forces, torques and power reached during the process.

As shown in the aforementioned literature, the cooling compressed air has been also applied in the machining of composites with polymeric matrix and different reinforcements as carbon fiber [12] or glass fiber [13], and an improved performance has been reported. Nevertheless, studies towards the drilling of reinforced polyether-ether-ketone with 30% glass fiber (PEEK-GF30) have not been found in the scientific literature. The novelty in this study is the analysis of its behavior during drilling processes, the most important process in the assembly as the previous operation to riveting and tapping. This material can withstand very low temperatures maintaining a stable behavior [14], therefore the machining of this material with cooling compressed air can be adequate as its mechanical and chemical properties are not expected to modify significantly during the process. Due to its extended use in the industry, its machinability has been studied, finding that with an adequate selection of cutting parameters and tools, it is a material that can be widely used in industry [15].

This environmentally friendly procedure can be contrasted evaluating the main variables of drilling under specific cutting conditions. Variables such as thrust forces should be low to improve process stability, at least in drilling operations; the literature shows that a homogeneous variation of thrust forces does not exist with respect to cutting conditions, at least in different types of composites such as glass fiber reinforced PEEK [16], fibre metal laminates [17], magnesium matrix based silicon carbide and graphene nanoplatelets (Mg/SiC/GNPs), hybrid magnesium matrix composite [18] or wood-based composite of medium density fiberboard [19]. Nevertheless, the energy consumed during the process could be a key factor to stablish the most sustainable procedure, and also the material removed rate (MRR) in order to evaluate the process efficiency. In the study carried out by Davim and Reis [20], the precision of the hole was considered as a variable to measure surface quality, so the diameter of the hole is another variable to study. Although the surface roughness is another variable that determines the hole quality, its influence in inner holes depends on the hole functionality; in this study, the evaluation of roughness surface it is irrelevant as an internal threading operation is expected to approach; its evaluation is important in other further operations such as riveting, however this characteristic can be corrected in other finishing operations, even with the same tool. Thus, the main variable to be accepted as an adequate dimensional accuracy of the holes is their dimension, and moreover it can be influenced by the chip evacuation and thermal expansion of the matrix occurring during the cutting process due to the lack of lubricant, as can be seen in materials based on multilayer metallic and/or composite stacks such as comprising titanium stacks, carbon fibre reinforced plastics (CFRPs) and aluminium [21], functionally graded composite, carbon/epoxy and glass/epoxy composite [22] or carbon fiber-reinforced plastic composite [23]. The reduction of the temperature in the cutting area could avoid this effect.

Taking into account the above-mentioned points, the objectives of this paper are the following: (i) to find the cutting conditions more environmentally friendly, with less energy consumption, in the drilling of reinforced PEEK with glass fiber at 30% with cooling compressed air by vortex tube, and (ii) to establish a balance between environmental conditions and adequate process performance.

## 2. Materials and Methods

The methodology used in this work combines experimental and statistical procedures.

### 2.1. Experimental Procedure

Drilling tests were carried out in a CNC vertical machining center, Manga Tongtai TMV-510 (Kaohsiung Hsien, Taiwan). The drilling operations were performed on plates of PEEK-GF30 (Gapi, Bergamo, Italy), with thickness of 6.5 mm, and a coefficient of linear thermal expansion of 30 × 10^−6^ m/m·K).

The drill bits employed were provided by FMT Tooling Systems Company (Trofa, Portugal); they had a diameter of 6 mm and are made of solid carbide with coating of zirconium oxide. Their material and geometrical characteristics are described in Table 1. Note that, as the point angle of drill was 140°, the corresponding cutting length was 7.592 mm. The selection of cutting tool was made based on the results of the analysis of variables, such as, for example, the energy or CO_2_ emissions [24] and preliminary tests. In drilling processes, the use of coating of zirconium oxide provides a good behavior of tool respect to the tool wear, the cutting forces and the chip flow [25]. A design of experiments (DOE) was used to optimize the resources. In particular, a three-level factorial design with three replicates was employed to analyze the influence of the three factors: feedrate, spindle speed and environmental temperature, on the response variables: thrust force, energy required for the drilling operation, material removed rate and drill bit diameter. This was, in total, 81 drilling tests. Later on, in the Section 2.2 statistical procedure, a more detailed description of the statistical analysis method is included.

Values of spindle speed (*N*), from 5000 to 7000 rpm and values of feedrate (*f*) from 0.5 to 1 mm/rev were applied. The drilling tests were carried out at temperatures (*T*) from −22 to 22 °C. The speed and the feed were chosen taking into account previous tests, information exchanged with the tool supplier, characteristics of the drill bits and high performance. Besides, similar ranges can be found in the literature [15]. Note that the feedrate was very high and this fact allowed quickly increasing the MRR. Firstly, drilling tests were performed at the room temperature that was 22 °C and secondly, drilling tests were carried out with the cooling compressed air system at 0 and −22 °C; thus, three specimens were used, one for each temperature. For the measurement of the temperature, an infrared pyrometer, Optris, was used; in this way, the temperature was monitored along the drilling process. The cooling was achieved by means of a Ranque–Hilsch vortex tube with two outlets of cold fluid, Dual Nozzle CAG Vortec model. This cooling technology allows obtaining the most improved outcomes when the nozzle number is 2, due to a more homogeneous distribution of cooling [26]. In other machining processes, with regard to this technology it is underlined that cutting forces and the power required in the titanium turning could be reduced with the application of vortex tube [27]. Similar findings were obtained by other studies with respect to flank wear and surface roughness towards the machining of aluminum alloys [28].

For the application of the cooling compressed air, it was necessary that the tube received the compressed air with a pressure of 0.8 MPa. To achieve temperatures of 0 and −22 °C, the compressed air had to flow for 8 and 10 min respectively, along vortex tube, before impinging on the drill bit; this step was very important to avoid the formation of ice on the drill bit during the drilling process. The nozzles of vortex tube were positioned at a distance of 30 mm, approximately, to achieve these temperatures.

**Figure 1:**
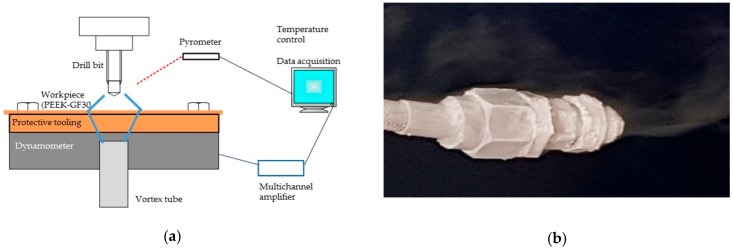
Machining process and data collection: (**a**) Scheme of assembly to drilling process and data capture; (**b**) Outlet of cold fluid after 8 minutes, with ice in the exterior.

The thrust force and the torque on the drilling direction were calculated directly during the monitoring of the drilling process. A piezoelectric dynamometer type Kistler 9257B connected to multichannel amplifier type Kistler 5070A was used (see Figure 1). The data were processed by DasyLab software (version 9.0, Measurement Computer, Norton, USA) [29]. The torques were taken to calculate the energy according to Equation (1), derivated of the expression used by Li et al. in the drilling of titanium alloys [30].

(1) $$E=\int_{l}^{}Ft\times dl+\int_{l}^{}{\left(\frac{2\times \pi \times To}{f}\right)}_{z}\times dl\;,$$

where *E* is the energy required to drill a hole in J, *Ft* is the thrust force in N, *l* is the drilling length in m, *To* is the torque respect Z axis in N·m, and *f* is the feedrate respect Z axis, in m/rev.

In the drilling process, the material removed rate, in mm^3^/s, is identified according to Equation (2) [31],

(2) $$MRR=\left(\pi \times Di^{2}\times F\right)/\left(4\times 60\right)$$

where *Di* is the drill bit diameter in mm, *F* is the feedrate in mm/min.

**Figure 2:**
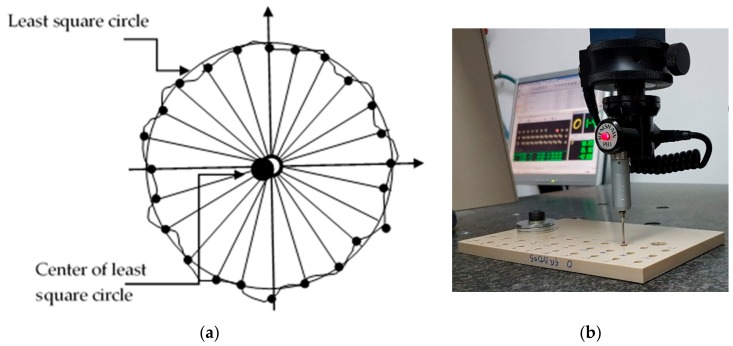
Measurement process of diameters: (**a**) Scheme of determination of center and circle of least square; (**b**) Coordinate measuring machine, during the measurement process of diameters.

To control the diameters quality, measurements were taken on 24 points along the circumference of the hole (as considered a suitable number [32]) by a coordinate measuring machine, Mitutoyo BX 303. The least square circle method was used to calculate the diameter measurements. Each measurement was repeated three times. The procedure of the measurement method was developed according to ISO 4291 standard [33]. The surface points were measured using a ball point stylus with a diameter of 1.6 mm. The data obtained were fitted by a Gaussian filter with 50% cut off. The outcomes were treated by Geopak-Win MCC software. Figure 2a shows a scheme of the location of center and circle of measured point after applying least square circle technique. Figure 2b shows the coordinate measurement machine.

### 2.2. Statistical Procedure

The statistical procedure focused on the application of response surface methodology when several responses should be simultaneously optimized, through a multi-objective method, in particular the desirability function, which allows finding a common objective for variables with different sub-objectives.

The experimental data collected were statistically analyzed, in particular, by the Response Surface with three-level factorial design, 3 × 3 in this case, and using Statgraphics software [34]. The Analysis of Variance (ANOVA) was carried out for the variables thrust forces, energy and MRR, meanwhile the diameter was used to evaluate the quality of the holes. With ANOVA analysis, the significant factors can be identified in a particular confidence interval [33]. The ANOVA of each variable is shown by tables, where the sum of squares or variance of the observations, the degrees of freedom (Df), the mean square, the F-ratio obtained from Fisher–Snedecor distribution and its probability associated (P-value) are represented; values of means squares are calculated dividing the sum of squares by its associated degrees of freedom. In this case, the confidence interval considered was 90%, adequate in manufacturing environments; therefore, a P-value less 0.1 denoted that the factor was significant using an ANOVA analysis. Although a 95% confidence interval is usually common in ANOVA studies, the election of 90% allows increasing the range of significance in the energy variable, which is dependent of other two variables, as it can be observed in Equation (1). Moreover, from a statistical perspective, this interval is adequate [35]. The percentage of contribution of the significant factors to variability was determined by dividing the sum of squares for the factor by sum of squares total. An analysis of means (ANOM) was also carried out to determine if there are differences between the means of each variable for the values considered; this analysis was performed through Tukey-honestly significant difference (HSD) test, which allows identifying what means are different at a confidence interval of 95% [36].

Once an ANOVA study and an ANOM study were developed, regression models were defined based on the surface response [36], and finally, the desirability function was defined. The desirability function, *d_i_*, was defined by Derringer and Suich [37], with different expressions according to the objective of the study variable. In this paper, there are, a variable to maximize, the MRR, and two variables to minimize, the thrust force and the energy. When the variable must be maximized, Equation (3) represents this option [37]:

(3) $$d_{i}\left({\hat{y}}_{i}\left(x\right)\right)=\left\{\begin{array}{c} 0\;\;\;\;\;\;\;\;\;\;\;\;\;\;\;\;\;\;\;\;\;\;\;\;\;\;\;\;if\;\;{\hat{y}}_{i}\left(x\right)<L_{i}\\ {\left({\hat{y}}_{i}-L_{i}/U_{i}-L_{i}\right)}^{s}\;\;\;if\;L_{i}\leq {\hat{y}}_{i}\left(x\right)\leq U_{i}\\ 1\;\;\;\;\;\;\;\;\;\;\;\;\;\;\;\;\;\;\;\;\;\;\;\;\;if\;\;\;{\hat{y}}_{i}\left(x\right)>U_{i} \end{array}\right.,$$

where *ŷ_i_* is *i*th estimated response of the variable (thrust force, energy, MRR), *L_i_* is lower acceptable value, *U_i_* is the upper acceptable value and *s* is the weight. Equation (4) represents the desirability function when the response must be minimized, being *t* the weight [37].

(4) $$d_{i}\left({\hat{y}}_{i}\left(x\right)\right)=\left\{\begin{array}{c} 1\;\;\;\;\;\;\;\;\;\;\;\;\;\;\;\;\;\;\;\;\;\;\;\;\;\;\;\;\mathrm{if}\;\;{\hat{y}}_{i}\left(x\right)<L_{i}\\ {\left(U_{i}-{\hat{y}}_{i}/U_{i}-L_{i}\right)}^{t}{\;\mathrm{if}\;L}_{i}\leq {\hat{y}}_{i}\left(x\right)\leq U_{i}\\ 0\;\;\;\;\;\;\;\;\;\;\;\;\;\;\;\;\;\;\;\;\;\;\;\;\;\mathrm{if}\;\;\;{\hat{y}}_{i}\left(x\right)>U_{i} \end{array}\right.,$$

Therefore *d (ŷ_i_*(x)) takes, uniquely, a value between 0 and 1 when it transforms a response into free-scale. The global desirability (*D*) can be determined through a weighted geometric mean by Equation (5) [37]:

(5) $$\;D={\left(\prod_{i=1}^{n}d_{i}^{\mathrm{ri}}\right)}^{1/\sum \mathrm{ri}},$$

where *n* is the number of variables and *ri* is the impact value. Thus, *D* takes also takes values between 0 and 1, and its optimum consists to maximize it. The values of the weights and impact can be fixed by the authors. In case of using Statgraphics software, the values can vary between 1 and 5. This range allows to establish the usual values in manufacturing environments, in particular in machining processes, e.g., in tapping operations [13] or in turning operations [38].

## 3. Results and Discussion

In Table 2, the experimental results obtained are detailed: thrust forces, energy, MRR and input diameter of holes. The values are the result of calculating the means of three values because the reproducibility of the measurements was very high. Outcomes from forces or energy show the influence of the feedrate, in a way that at higher feedrates, a reduction of the values of these variables were obtained, compared to other experimental data [16], which could be explained by the fact that the flute helix angle of tools improves chip evacuation. A low variability can be observed in data from input diameters; in all tests except in test 21, diameters slightly lower than the nominal drill bit (6 mm) were obtained. The reason could be the improved chip evacuation and the lack of thermal expansion during the drilling tests.

### 3.1. Analysis of Experimental Results

This subsection is developed to explore the experimental results; the influence of the considered cutting factors (feedrate, spindle speed and environmental temperature) are analyzed.

#### 3.1.1. Thrust Forces

The ANOVA analysis for thrust forces (see Table 3) shows that the significant main factors were environmental temperature (T), spindle speed (N) and feedrate (f), and the significant interactions are T^2^, T × N, N^2^ and T^2^ × N in a confidence level of 90%. The most influential main factor was the spindle speed, with a contribution of 24.82%, as was expected due to the influence of the incidence of the drill bit on the plate. As can be seen, the contribution of temperature as main factor (first or second order) was 25.31% and taking into account its interactions with spindle speed, T × N and T^2^ × N, their contribution reached 49.12%. Thus, the temperature influence was clear; in fact, in Table 2 it can be observed, as less desirable results were obtained at temperatures close to 0 °C; similar results were approached in the tapping of PA66-GF30 as shown in the study carried out by Domingo et al. [13].

On the other hand, the ANOM study and the Tukey-HSD test reveal that the pairwise means were significantly different from each other at 95% confidence level, with respect to feedrate (statistically significant differences between pairwise means, 0.5–0.75: −7.67, 0.5–1: −19.19 and 0.5–1: −11.53), spindle speed (statistically significant differences between pairwise means, 5000−6000: 2.71, 5000−7000: 27.53, and 6000−7000: 24.82), and enviromental temperature (statistically significant differences between pairwise means: −22–0: –47.28, −22–22: −33.65, and 0–22: 14.23). This denotes that the thrust force showed different behavior in each of the selected cutting speeds, feedrates and temperatures. 

According to the above, thrust forces obtained in drilling processes depended strongly on the enviromental temperature.

#### 3.1.2. Energy

From the ANOVA analysis for energy (Table 4), the factors N^2^, f^2^, T × N × f, T × f^2^ and N × f^2^ (not significant) were eliminated in order to achieve a more improved adjustment between the factors. Feedrate and T^2^ erre the significant factors at 90% confidence level, with an influence of 9.19% and 6.78%, respectively. Although the effect of these factors was lower than those considered for thrust force, the environmental temperature and cutting conditions reappeared.

In this case, from Tukey-HSD test it was obtained that the pairwise means were significantly different from each others at 95% confidence level, with respect to feedrate (statistically significant differences between pairwise means, 0.5–0.75: 4.10, 0.5–1: 6.98 and 0.5–1: 2.86), spindle speed (statistically significant differences between pairwise means, 5000–6000: −0.56, 5000–7000: 0.08, and 6000-7000: 0.67), and temperature (statistically significant differences between pairwise means: −22–0: −3.88, −22–22: −2.97, and 0–22: 0.91). This implies that the energy showed a different behaviour in each of the selected spindle speeds, feedrates and temperatures.

#### 3.1.3. Material Removed Rate

The ANOVA analysis for MRR can be seen in Table 5. Although the data from different temperatures were included in the study, the significant factors were N, f, N × f and f^2^, being the most influential factor on the feedrate (f) with a percentage of 44.9%, as was expected despite f^2^ having a very low influence. Note that the feedrate considered was very high.

From Tukey-HSD test it was obtained that the pairwise means were significantly different from each other at 95% confidence level, with respect to feedrate (statistically significant differences between pairwise means, 0.5–0.75: −706.85, 0.5–1: −1431.71 and 0.5–1: −706.86) and spindle speed (statistically significant differences between pairwise means, 5000–6000: −353.43, 5000–7000: −706.86, and 6000−7000: −353.43). Obviously, there were no statistically significant differences between pairwise means with respect to temperature because the MRR was independent of it, with differences as: −22–0: 0, −22–22: 0, and 0–22: 0. This implies that the energy showed a different behaviour in each of the selected spindle speeds and feedrates.

#### 3.1.4. Input Diameter

**Figure 3:**
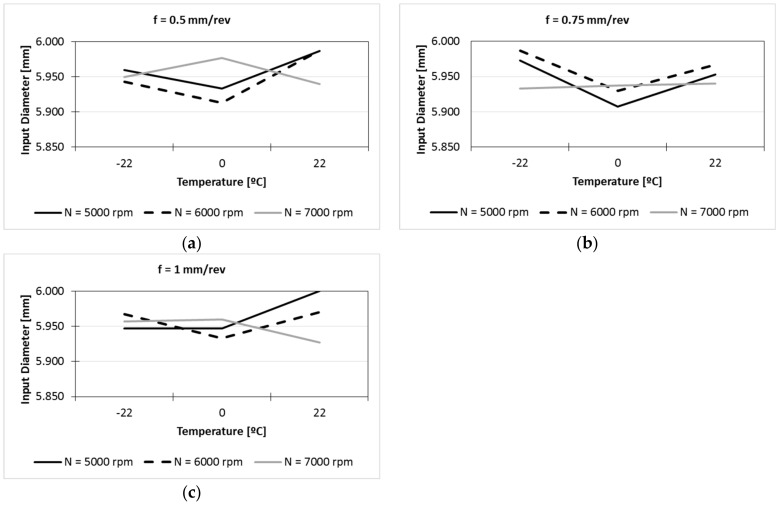
Input diameter executed at different temperatures and spindle speed, and with: (**a**) feedrate of 0.5 mm/rev; (**b**) feedrate of 0.75 mm/rev; (**c**) feedrate of 1 mm/rev.

As has been mentioned before, the measurements of input diameters were carried out as quality control evaluation; if measurements were correct the experimental tests could be considered valid. Otherwise, the tests could not be taken out and the experimental data would have to be discarded. In Table 2 can be seen that the greater difference between diameters was 0.093 mm (0.013 mm at 22 °C, 0.070 mm at 0 °C and 0.054 mm at −22 °C), being adequate cooling temperaturesfor different spindle speeds at feedrates of 0.5 mm/rev (Figure 3a), 0.75 mm/rev (Figure 3b) and 1 mm/rev (Figure 3c). In these figures, larger undersized holes could be observed at 0 °C with spindle speed of 5000 and 6000 rpm. However, the difference between them was very low, and it did not seem relevant. Perri et al. [11] found that the effect of the cooling system and the flow air on the displacement of the tool centre point was less intense; this can explain the data obtained in the diameters, and the undersized holes obtained. 

### 3.2. Response Surface

Taking into account, uniquely, the significant main factors and interactions, and with a coefficient of determination, R^2^, superior to 70% in all of the cases, the regression equations, Equations (6)–(8), are the following:

(6) $$Ft=83.727-5.662\times T-0.0415\times N+223.276\times f-0.455\times T^{2}+\;+0.00193\times T\times N+0.0000059\times N^{2}+0.0\times T^{2}\times N,$$

(7) $$E=-129.475+195.038\times f-0.01164\times T^{2},$$

(8) $$MRR=0.05-0.14\times f+0.47124\times N\times f+0.08\times f^{2},$$

**Figure 4:**
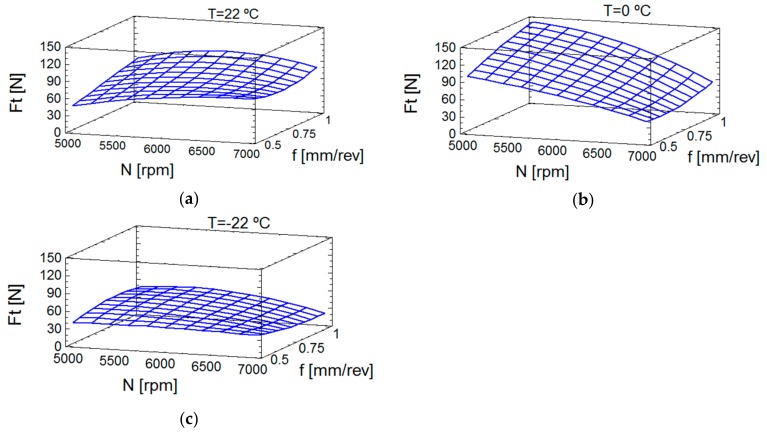
Estimation of response surface for thrust forces: (**a**) Temperature of 22 °C; (**b**) Temperature of 0 °C; (**c**) Temperature of −22 °C.

The effect of the temperature on thrust forces can be seen in Figure 4. The influence of spindle speed and the feedrate was lower at room temperature (Figure 4a) than at 0 °C (Figure 4b). Finally, it is remarkable that, at −22 °C (Figure 4c), the thrust forces were lower and less dependent of cutting conditions and the lowest values were reached. Figure 4 plots the values of Equation (6), also, the evolution of Ft and its trend, with continuous values. The evolution of forces at 0 °C deserves special mention, that showed the force decreased with the increase of spindle speed; the friction seemed to increase at this temperature, though this effect was diminished at higher spindle speeds; in this case, the feedrate more convenient is the lowest value. However, at −22 °C, the friction phenomenon did not seem to be affected, which could be explained because of greater influence of the air pressure at low temperature. Note that, as mentioned in Section 1, PEEK-GF30 maintains a stable behavior at low temperatures [14], which is very important because it prevents the modification of the material properties at these temperatures.

**Figure 5:**
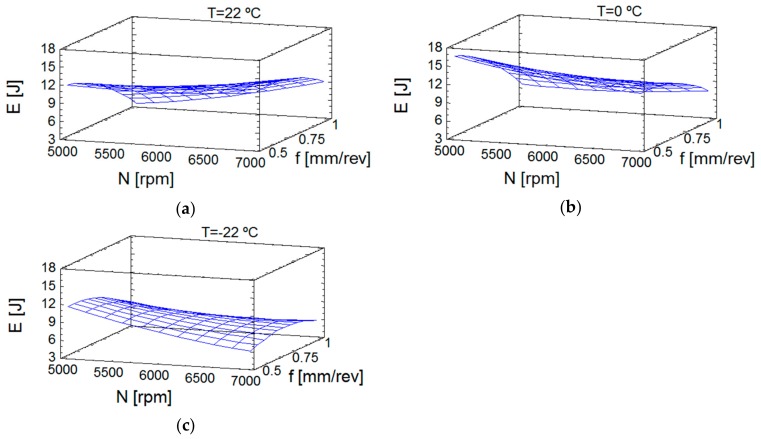
Estimation of response surface for energy: (**a**) Temperature of 22 °C; (**b**) Temperature of 0 °C; (**c**) Temperature of −22 °C.

The estimation of response surface for energy can be seen in Figure 5. At 22 °C (Figure 5a) and 0 °C (Figure 5b) the distribution of the energy obtained was more uniform. At −22 °C (Figure 5c), the highest spindle speeds provided a similar behavior to that in thrust forces at this temperature, despite the greater influence of the torque in the calculation of the energy consumed during drilling [16]. The explanation can be the same as found for the forces, given the dependence of the cutting conditions along the cutting length during drilling (see Equation (1)). To clarify this point, in Figure 5 the values of Equation (7), the evolution of energy and its trend are plotted. Dry machining (at 22 °C) is an option with more energy consumption, maybe because there is a heating in the cutting area.

**Figure 6:**
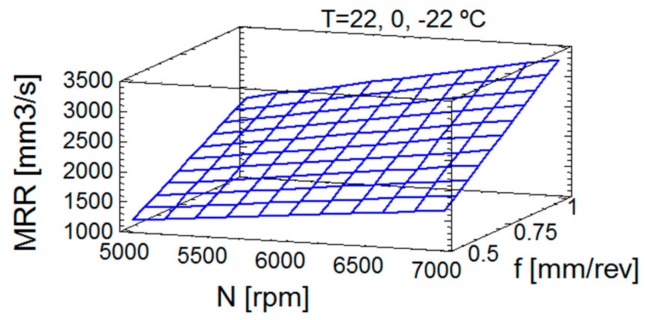
Estimation of response surface for MRR, at 22 °C, 0 °C and −22 °C.

Figure 6 shows the estimation of response surface for MRR, with the clear and known influence of cutting conditions. This representation of Equation (8) allows the representation of the evolution of MRR, its increase with the cutting conditions, as expected, and its independence of temperature, as can be observed in Equations (2) and (8). In Figure 6, from the last equation MRR values are plotted respect to cutting conditions, spindle speed and feedrate.

### 3.3. Multiple Response Surface Optimization

The multiple response surface optimization is represented in Table 6; in this Table, the goal of each variable and the parameters values are shown, lower values of variables (*L_i_*), upper values of variables (*U_i_*), weights considered in the desirability function (*s* and *t*), and impact. The values taken for weights and impact avoid that some variables influenced more than others in the results, and besides they are used to optimize the manufacturing processes, in particular in machining operations [13,38]. As can be seen in Table 6, the weights and the impact were the same for each variable, 1 for weights and 3 for impact; in this manner, all variables were compensated. The lower and upper values of each variable were taken from Table 2; the Ft values corresponded to −22 °C, 7000 rpm and 0.75 mm/rev (lowest values) and 0 °C, 5000 rpm and 1 mm/rev (uppest value); the Energy values corresponded to −22 °C, 6000 rpm and 1 mm/rev (lowest value) and 22 °C, 7000 rpm and 0.5 mm/rev (uppest value); and MRR corresponded to any temperature, and 5000 rpm and 0.5 mm/rev (lowest value) and 7000 rpm and 1 mm/rev (uppest value). This indicates the importance of the application of a multi-objective method to seek a common objective when the goals are different and when the minimum and maximum values of each variable correspond to different cutting conditions. 

**Figure 7:**
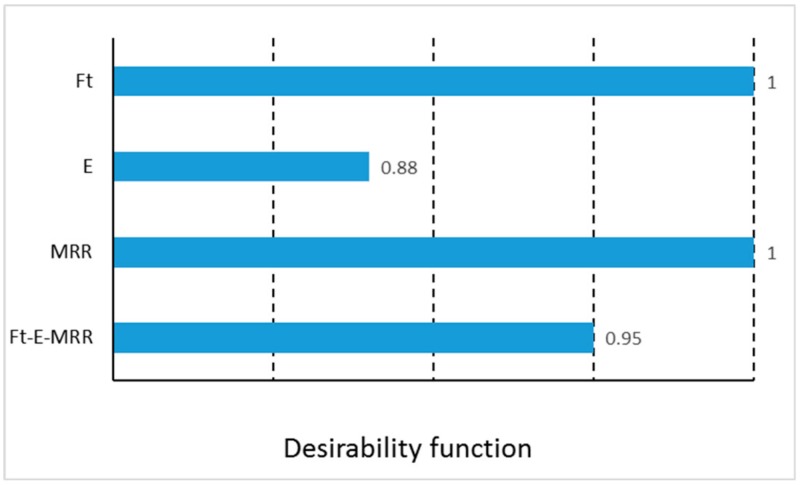
Desirability function.

Figure 7 shows the values of desirability function for thrust force, energy, MRR and Ft-E-MRR; while that the maximum value (1) was achieved for thrust forces and MRR, the minimum value was for the energy, that obviously it was noted in the final value for the combined variable Ft-E-MRR. While with Ft and MRR, the maximum desirability was achieved, the energy showed a value of 0.88. This value can be considered normal due to the dependence of different variables on its calculation (see Equation (1)).

**Figure 8:**
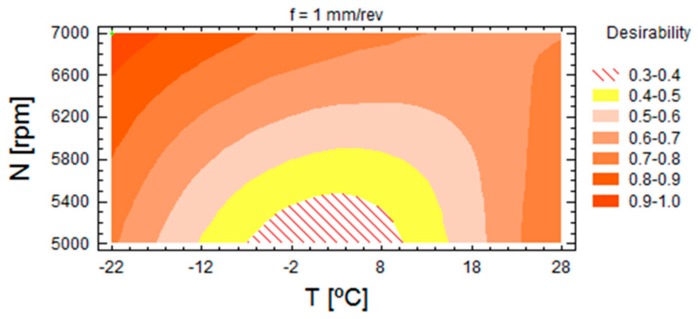
Contours of estimated response surface for f = 1 mm/rev.

The optimization allowed finding the maximum desirability at −22 °C, feed of 1 mm/rev and 7000 rpm; under these conditions, the optimum values were 22.93 N for thrust force, 4.2 J for energy and 3298 mm^3^/s for MRR. Figure 8 shows the contours of estimated response surface. From this graphic it can be observed that the area of major desirability was located at a temperature of −22 °C and at high spindle speed, considering the thrust force, the energy and the MRR. Note that the optimal parameters allowed machining at high cutting conditions at −22 °C. This possibility can increase the potential use of machining at low temperatures by avoiding thermal expansion of the matrix of composite materials. This balance combines objectives of sustainability and efficiency.

## 4. Conclusions

The drilling of reinforced PEEK plates with drills of coating zirconium oxide and diameter of 6 mm was analyzed at different cutting conditions and environmental temperatures, employing cooling compressed air by a Ranque–Hilsch vortex tube. Experimental data from spindle speed, feedrate, temperature and input diameter of holes were measured, and a statistical study was developed. 

The Response Surface methodology with three-level factorial design was applied to optimize, simultaneously, several responses through the desirability function, in order to find the significant factors (spindle, speed, feed rate and temperature) and their interactions on the variables such as thrust force, energy and MRR, the relationships between factors and variables, and the value of desirability function. Thus, the response surface methodology should be optimized. 

From the results of this experimental and statistical study, conclusions can be summarized as:

- The environmental temperature (in first, second degree) and its interaction with spindle speed is a significant factor for thrust forces and for energy (in first degree).
- The maximum desirability function was found at highest cutting conditions (7000 rpm and 1 mm/rev) and at −22 °C. The implementation of compressed cooling air provides a balance between the optimal conditions for the analyzed variables.
- Cooling compressed air can be an environmentally friendly procedure that reduces the energy consumption, and besides, it can be compatible with high cutting conditions, which facilitates the high performance of machining processes.
- Finally, oversized holes can be avoided at the conditions used in this study.

Future researches can address lower temperatures, avoiding temperatures close to 0 °C which do not improve the performance with respect to dry conditions at room temperature. Moreover, in future developments, the application of cryogenic drilling on this material can be considered due to the improved outcomes obtained at low temperatures. In addition, the tool wear is proposed to be a factor to analyze.

## Figures and Tables

**Table 1:** Drill bits characteristics.

	Material	Coating	Point Angle	Flute Helix Angle	Number of Cutting Edges	Nominal Tolerance
Drill bit	Solid carbide	Zirconium oxide	140°	15°	2	h7

**Table 2:** Experimental results.

No. Test	T (°C)	N (rpm)	f (mm/rev)	Ft (N)	E (J)	MRR (mm^3^/s)	Di (mm)
1	−22	5000	0.5	47.12	10.67	1178.10	5.960
2	0	5000	0.5	81.32	12.83	1178.10	5.933
3	22	5000	0.5	59.15	16.28	1178.10	5.987
4	−22	6000	0.5	38.68	15.47	1413.72	5.943
5	0	6000	0.5	85.27	19.54	1413.72	5.913
6	22	6000	0.5	57.05	12.66	1413.72	5.987
7	−22	7000	0.5	31.05	7.54	1649.34	5.950
8	0	7000	0.5	49.26	8.56	1649.34	5.977
9	22	7000	0.5	75.16	19.93	1649.34	5.940
10	−22	5000	0.75	47.72	9.08	1767.15	5.973
11	0	5000	0.75	129.24	13.7	1767.15	5.907
12	22	5000	0.75	58.31	7.82	1767.15	5.953
13	−22	6000	0.75	51.52	7.25	2120.57	5.987
14	0	6000	0.75	96.75	9.8	2120.57	5.930
15	22	6000	0.75	80.4	8.83	2120.57	5.967
16	−22	7000	0.75	26.57	4.85	2474.00	5.933
17	0	7000	0.75	30.70	14.6	2474.00	5.937
18	22	7000	0.75	71.81	10.65	2474.00	5.940
19	−22	5000	1	45.70	5.90	2356.19	5.947
20	0	5000	1	156.66	9.50	2356.19	5.947
21	22	5000	1	70.15	2.91	2356.19	6.000
22	−22	6000	1	47.58	2.67	2827.43	5.967
23	0	6000	1	103.32	8.39	2827.43	5.933
24	22	6000	1	110.36	9.46	2827.43	5.970
25	−22	7000	1	27.69	6.26	3298.67	5.957
26	0	7000	1	56.65	7.69	3298.67	5.960
27	22	7000	1	78.68	7.92	3298.67	5.927

**Table 3:** Analysis of Variance for *Ft*.

Source	Sum of Squares	Df	Mean Square	F-Ratio	P-Value
T	913.809	1	913.809	5.18	0.0461
N	6461.76	1	6461.76	36.63	0.0001
f	1524.32	1	1524.32	8.64	0.0148
T^2^	5676.25	1	5676.25	32.18	0.0002
T × N	724.941	1	724.941	4.11	0.0701
T × f	338.247	1	338.247	1.92	0.1962
N^2^	732.762	1	732.762	4.15	0.0689
N × f	498.843	1	498.843	2.83	0.1235
f^2^	22.4396	1	22.4396	0.13	0.7287
T^2^ × N	5476.74	1	5476.74	31.05	0.0002
T^2^ × f	466.632	1	466.632	2.65	0.1349
T × N^2^	29.6117	1	29.6117	0.17	0.6906
T × N × f	3.83645	1	3.83645	0.02	0.8857
T × f^2^	52.1043	1	52.1043	0.30	0.5987
N^2^ × f	128.633	1	128.633	0.73	0.4131
N × f^2^	139.122	1	139.122	0.79	0.3953
Total residual	1763.86	10	176.386		
Total	26,038.0	26			

**Table 4:** Analysis of variance for *E*.

Source	Sum of Squares	Df	Mean Square	F-Ratio	P-Value
T	5.15227	1	5.15227	0.53	0.4783
N	4.47207	1	4.47207	0.46	0.5084
**f**	**46.5409**	**1**	**46.5409**	**4.78**	**0.0451**
**T^2^**	**34.3523**	**1**	**34.3523**	**3.53**	**0.0800**
T × N	28.49	1	28.49	2.92	0.1079
T × f	7.88941	1	7.88941	0.81	0.3825
N × f	4.45301	1	4.45301	0.46	0.5093
T^2^ × N	6.12562	1	6.12562	0.63	0.4402
T^2^ × f	7.7748	1	7.7748	0.80	0.3858
T × N^2^	2.828	1	2.828	0.29	0.5980
N^2^ × f	9.68247	1	9.68247	0.99	0.3347
Total residual	146.171	15	9.74475		
Total	506.569	26			

**Table 5:** Analysis of variance for material removed rate (*MRR)*.

Source	Sum of Squares	Df	Mean Square	F-Ratio	P-Value
T	0.0	1	0.0	0.0	1.0000
**N**	**449,686.0**	**1**	**449,686.0**	****	**0.0000**
**f**	**1.79872 × 10^6^**	**1**	**1.79872 × 10^6^**	****	**0.0000**
T^2^	0.0	1	0.0	0.0	1.0000
T × N	0.0	1	0.0	0.0	1.0000
T × f	0.0	1	0.0	0.0	1.0000
N^2^	0.0	1	0.0	0.0	1.0000
**N × f**	**166,550.0**	**1**	**166,550.0**	****	**0.0000**
**f^2^**	**0.00015**	**1**	**0.00015**	**39,607.88**	**0.0000**
T^2^ × N	0.0	1	0.0	0.0	1.0000
T^2^ × f	0.0	1	0.0	0.0	1.0000
T × N^2^	0.0	1	0.0	0.0	1.0000
T × N × f	0.0	1	0.0	0.0	1.0000
T × f^2^	0.0	1	0.0	0.0	1.0000
N^2^ × f	0.0	1	0.0	0.0	1.0000
N × f^2^	0.0	1	0.0	0.0	1.0000
Total residual	3.78713 × 10^−8^	10	3.78713 × 10^−9^		
Total	1.14086 × 10^7^	26			

**Table 6:** Values considered in the desirability function.

Variable	Goal	L_i_	U_i_	*s*	*t*	Impact
Ft [N]	Minimize	26.57	156.66	1	–	3
E [J]	Minimize	2.67	19.93	1	–	3
MRR [mm^3^/s]	Maximize	1178.10	3298.67	–	1	3
